# Epitope-dependent effect of long-term cART on maintenance and recovery of HIV-1-specific CD8^+^ T cells

**DOI:** 10.1128/jvi.01024-23

**Published:** 2023-10-25

**Authors:** Nozomi Kuse, Hiroyuki Gatanaga, Yu Zhang, Takayuki Chikata, Shinichi Oka, Masafumi Takiguchi

**Affiliations:** 1 Division of International Collaboration Research and Tokyo Joint Laboratory, Department of Frontier Research, Joint Research Center for Human Retrovirus Infection, Kumamoto University, Kumamoto, Japan; 2 AIDS Research Center, National Institute of Infectious Diseases, Shinjuku-ku, Tokyo, Japan; 3 AIDS Clinical Center, National Center for Global Health and Medicine, Shinjuku-ku, Tokyo, Japan; Icahn School of Medicine at Mount Sinai, New York, USA

**Keywords:** HIV-1, CD8^+ ^T cells, cART, AIDS, epitope

## Abstract

The frequency of HIV-1-specific T cells is reduced and their functional ability remains impaired in people living with HIV-1 (PLWH) under combined antiretroviral therapy (cART). However, no studies have yet reported on T-cell responses to large numbers of well-characterized HIV-1 epitopes before and under cART within the same individuals. Therefore, how cART affects the maintenance of HIV-1-specific CD8^+^ T cells and which HIV-1-specific CD8^+^ T cells would be useful for curative treatment remain unclear. We investigated T-cell responses to 63 HIV-1 epitopes at pre-cART and under cART in 90 PLWH who received long-term cART. The frequency of T-cell responses to HIV-1 epitopes under long-term cART varied from 0% to 100%, indicating an epitope-dependent effect of long-term cART on HIV-1-specific CD8^+^ T cells. The magnitude of the responses under cART was 26.2% of that at pre-cART. The frequency of T-cell responses to protective epitopes under cART was higher than that to non-protective ones. T-cell responses to HIV-1 epitopes were detected at pre-cART in most non-AIDS HIV patients, whereas they were not detectable at pre-cART in half of the AIDS patients. Long-term cART enhanced the ability of HIV-1-specific T cells to proliferate *in vitro* in non-AIDS HIV patients, while it restored this ability of the T cells in AIDS patients. This study demonstrated that CD8^+^ T cells specific for large numbers of HIV-1 epitopes were maintained in an epitope-dependent fashion under long-term cART and implied that some HIV-1-specific T cells are still candidates as effector cells for curative treatment.

**IMPORTANCE:**

HIV-1-specific CD8^+^ T cells are anticipated to become effector cells for curative treatment using the “shock and kill” approach in people living with HIV-1 (PLWH) under combined antiretroviral therapy (cART). Previous studies demonstrated that the frequency of HIV-1-specific CD8^+^ T cells is reduced under cART and their functional ability remains impaired. These studies analyzed T-cell responses to a small number of HIV-1 epitopes or overlapping HIV-1 peptides. Therefore, the features of CD8^+^ T cells specific for HIV-1 epitopes under cART remain only partially clarified. Here, we analyzed CD8^+^ T cells specific for 63 well-characterized epitopes in 90 PLWH. We demonstrated that CD8^+^ T cells specific for large numbers of HIV-1 epitopes were maintained in an epitope-dependent fashion under long-term cART and that long-term cART enhanced or restored the ability of HIV-1-specific T cells to proliferate *in vitro*. This study implies that some HIV-1-specific T cells would be useful as effector cells for curative treatment.

## INTRODUCTION

Combined antiretroviral therapy (cART) has been shown to successfully suppress HIV-1 replication and has contributed to saving the lives of many people living with HIV-1 (PLWH). However, cART cannot completely eradicate HIV-1 due to the persistence of latently infected cells harboring replication-competent proviruses in individuals undergoing treatment (1 – 3). Current therapeutic strategies are directed toward eliminating latent viral reservoirs. One of them is the “shock and kill” approach, which is based on the reactivation of latent reservoirs with latency-reversing agents, followed by eradication of these cells by immunological responses mediated by HIV-1-specific CD8^+^ T cells and/or neutralizing antibodies (4, 5). Recent studies suggested that HIV-1-specific T cells play a key role in purging viral reservoirs in HIV-1-infected individuals undergoing cART (6, 7).

Many studies have reported that the number of HIV-1-specific T cells is reduced in PLWH receiving long-term cART compared with that in treatment-naïve PLWH (8 – 10). Functional analysis of HIV-1-specific CD8^+^ T cells showed that, in individuals undergoing cART, these T cells exhibited enhanced polyfunctionality and viral inhibition ability compared with those in progressors, but had reduced their abilities compared with those in controllers (11 – 13). Previous studies further demonstrated that the size of reservoirs was unchanged or slightly reduced during long-term cART (3, 14, 15). These results imply that HIV-1-specific CD8^+^ T cells during cART still lose functionalities that may be important for “shock and kill” approaches to achieve a cure. Taking previous studies together, it is suggested that latent reservoirs cannot be eradicated by HIV-1-specific CD8^+^ T cells naturally elicited under cART in PLWH. However, whether HIV-1-specific CD8^+^ T cells have the ability to eradicate the reservoirs if the functional ability of these T cells is enhanced *in vivo* remains unknown.

Previous studies analyzed CD8^+^ T cells specific for a small number of HIV-1 epitopes (9, 11, 16, 17) or T-cell responses to overlapping HIV-1 peptides under cART in PLWH (10, 18 – 20). Only a few studies directly compared T-cell responses to a small number of epitopes between before the initiation of cART and during cART within the same individuals (11, 16, 17). Therefore, whether T cells specific for all reported HIV-1 epitopes are maintained or newly elicited under conditions of weak antigen presentation, such as under long-term cART, remains unknown. From this situation, it is important to investigate CD8^+^ T cells specific for large numbers of HIV-1 epitopes before the initiation of cART and during cART within the same PLWH and then to clarify which epitope-specific CD8^+^ T cells were maintained in them. This analysis should clarify the HIV-1-specific CD8^+^ T cells maintained under long-term cART.

In the present study, we analyzed CD8^+^ T-cell responses to large numbers of well-defined HIV-1 epitopes and compared the frequency and function of these T cells between before the initiation of cART and during cART in the same individuals chronically infected with HIV-1 subtype B. We demonstrated T-cell responses to 56 epitopes under cART and showed that long-term cART enhanced the ability of HIV-1-specific T cells to proliferate *in vitro* in non-AIDS HIV patients and restored the frequency and function of HIV-1-specific T cells in AIDS patients. The present study provides an overview of HIV-1-specific CD8^+^ T cells at both epitope and donor levels and implied that CD8^+^ T cells specific for some HIV-1 epitopes will be candidates of effector T cells for curative treatment using the “shock and kill” approach.

## RESULTS

### T-cell responses to HIV-1 epitope peptides in chronically HIV-1-infected individuals who received cART for more than 2 years

**Fig 1:**
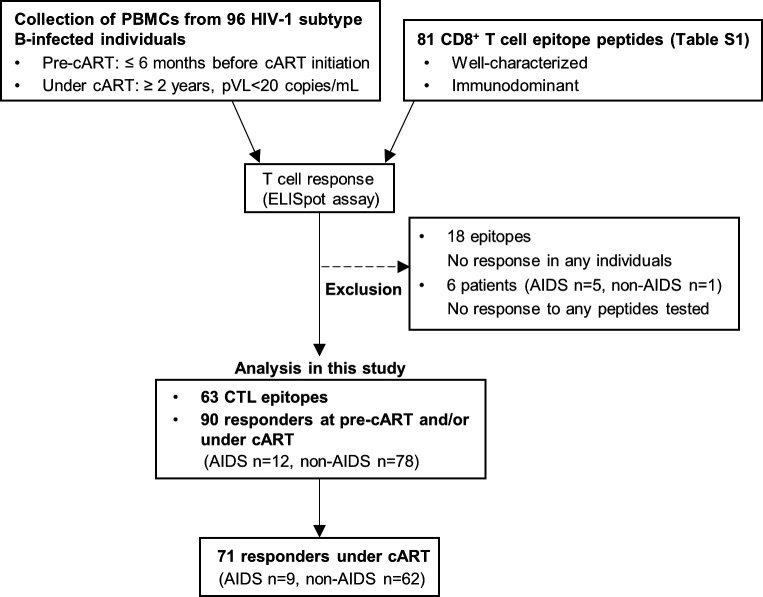
Profile of the present study.

We investigated T cells specific for HIV-1 CD8^+^ T-cell epitopes in peripheral blood mononuclear cells (PBMCs) from 96 PLWH who started cART at the chronic phase of infection and continued it for more than 2 years. Plasma viral load (pVL) was undetectable during cART in these individuals. PBMCs were collected less than 6 months before cART initiation (pre-cART) and at more than 2 years (2–8 years) after cART initiation (under cART). T-cell responses to 81 T-cell epitope peptides were analyzed by *ex vivo* ELISpot assay (Fig. 1; Table S1 in supplemental material). These CD8^+^ T-cell epitopes were selected based on the frequency of responders (>20%) among Japanese HIV-1-infected individuals analyzed in previous studies (21 – 46). These individuals were HLA (human leukocyte antigen)-typed and T-cell responses to HLA-matched epitope peptides were analyzed prior to and during cART. T-cell responses to 18 peptides (HLA-A*11:01-NefAK9, -EnvSK9, HLA-A*24:02-EnvFF9, -EnvRL8, -NefRW8, HLA-A*31:01-NefKR9, HLA-A*33:03-EnvVR10, -EnvVIR10, -EnvER8, -PolTR11, -GagMR9, HLA-B*35:01-PolNQY9, -GagHA9, HLA-B*51:01-EnvRI9, PolDL8, GagYI9, PolQI9, and HLA-B*67:01-GagNL11) were not found in any individuals tested. Six individuals did not have T-cell responses to the peptides tested. Therefore, the responses to 63 epitope peptides were analyzed in the 90 individuals. Responses to at least one epitope peptide were found under cART in 9 out of 12 AIDS patients at initiation of cART (AIDS patients) and in 62 out of 78 HIV-1-infected asymptomatic carriers at initiation of cART (non-AIDS HIV patients) (Fig. 1). Individuals who showed a positive response to a given epitope under cART but not at pre-cART were evaluated as responders under cART.

**Fig 2:**
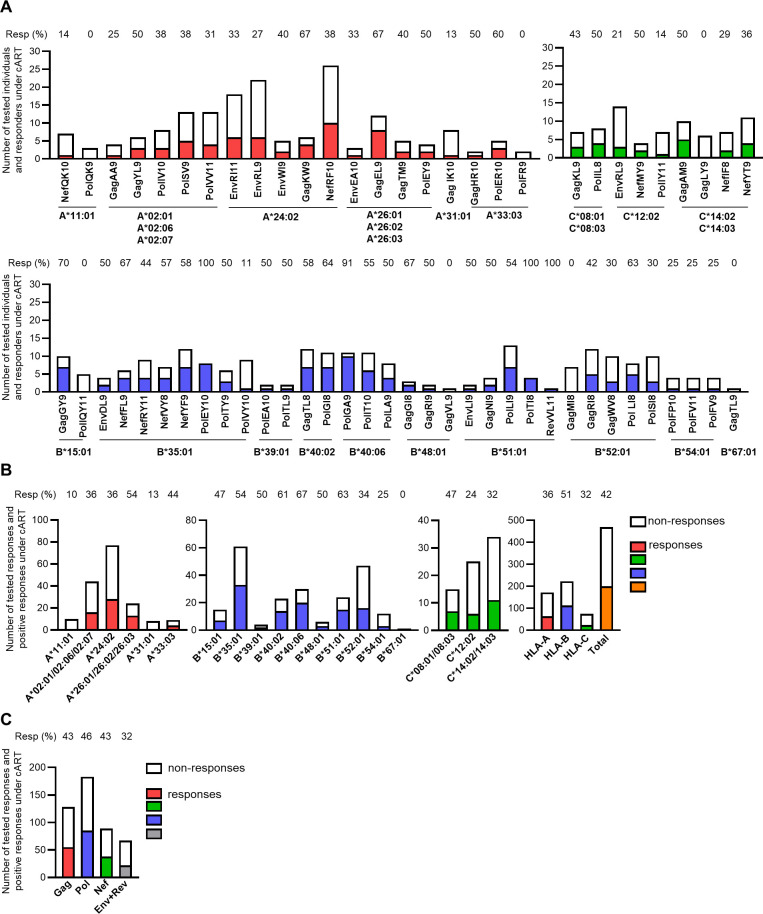
Frequency of responders to HIV-1 epitope peptide under cART. T-cell responses to 63 HIV-1 epitope peptides was tested by performing *ex vivo* ELISpot assay in 90 HIV-1 subtype B-infected individuals at pre-cART and under cART. (**A**) The numbers of non-responders (open bar) and responders (closed bar) to each HIV-1 epitope peptide under cART. (**B**) The number of tested responses and positive responses to HLA allele-restricted epitope peptides under cART. (**C**) The number of tested responses and positive responses to HIV-1 epitope peptides in each protein under cART. Resp, the frequency of responders (**A**), or positive responses (**B and C**). The frequency of T-cell responses to epitope peptide under cART was calculated as follows: (number of positive responses to epitope peptides under cART / number of tested responses to epitope peptides) × 100.

**Fig 3:**
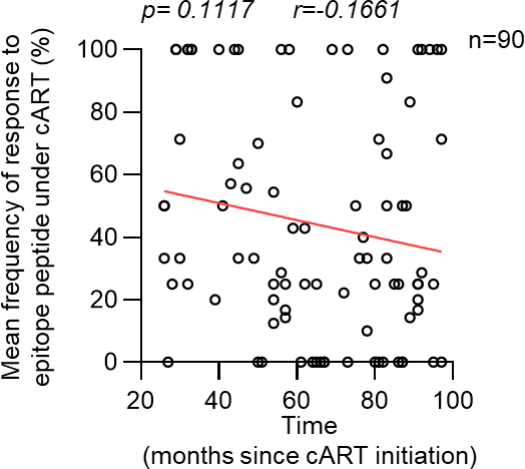
Effect of cART term on frequency of HIV-1-specific T-cell responses under cART. Mean frequencies of positive responses to HIV-1 epitope peptides under cART were analyzed in each individual. Correlation between mean frequencies of positive responses under cART and cART term (26 to 97 months) was statistically analyzed by Spearman’s correlation test. Each dot represents the mean frequency of all T-cell responses to epitope peptides under cART in each individual.

The frequency of responders to each epitope peptide under cART varied from 0% to 100% (Table S2 in supplemental material). Responses to seven epitope peptides were not found in any individuals tested, whereas responses to three epitope peptides were found in all individuals tested (Fig. 2A). These results suggest that the effect of long-term cART on HIV-1-specific CD8^+^ T cells is epitope-dependent. Positive responses to these epitope peptides under cART were found in 42.6% of the responses tested (Fig. 2B). The frequency of HLA-restricted T-cell responses was in the order of HLA-B-restricted > HLA-A-restricted > HLA-C-restricted T-cell responses (Fig. 2B), while that of T cells specific for epitopes in each HIV-1 protein was in the order of Pol-specific > Gag-specific = Nef-specific > Env/Rev-specific T-cell responses (Fig. 2C). We analyzed the correlation between cART duration (2–8 years) and the mean frequency of positive responses under cART at the donor level and found no significant correlation between them (Fig. 3), suggesting that cART duration does not influence T-cell responses to these epitope peptides between 2 and 8 years.

**Fig 4:**
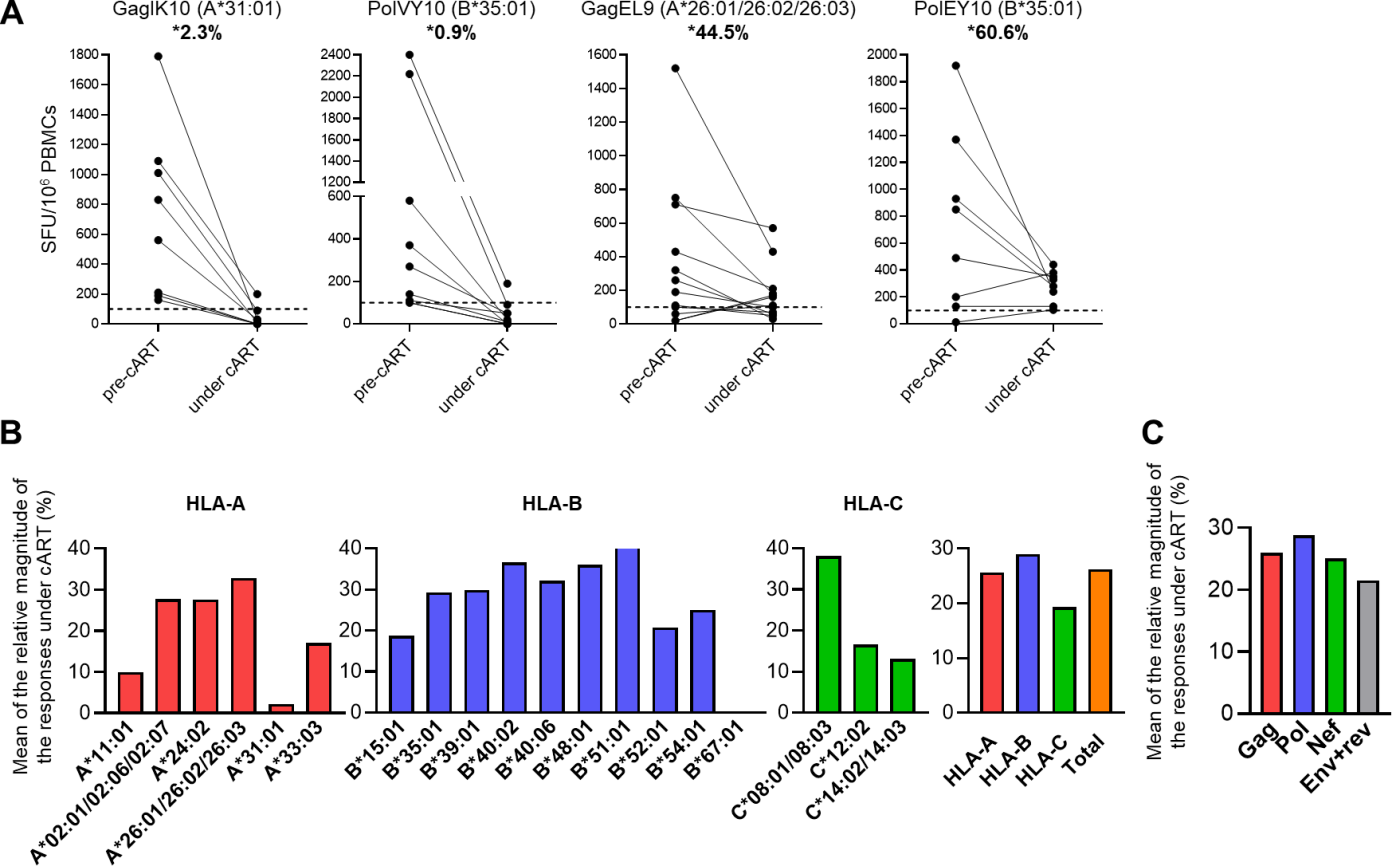
Magnitude of T-cell responses to each HIV-1 epitope peptide under cART (**A**). Representative cases of T-cell responses to epitope peptides at pre-cART and under cART. Responses to peptides at a concentration of 1 µM were analyzed by *ex vivo* IFN-γ (interferon-gamma) ELISpot assay. *Percentages of spot-forming unit (SFU) under cART versus that at pre-cART are presented in each figure. The dotted line at 100 SFU/10^6^ PBMCs indicates the threshold for a positive response. (**B and C**) Mean magnitude of T-cell responses to epitope peptides restricted by HLA-A, HLA-B, or HLA-C alleles (**B**) or to epitope peptides in HIV-1 Gag, Pol, Nef, or Env + Rev proteins (**C**) under cART versus at pre-cART.

We next compared the magnitude of the T-cell responses to each epitope peptide between under cART and pre-cART. The relative magnitude of the response to all tested epitope peptides (specific spot number for each HIV-1 epitope peptide under cART versus at pre-cART) is presented in Table 1. The mean relative magnitude of the response to the epitopes varied from 0% to 66.7% when the number of tested individuals was >2. Representative cases of high or low relative magnitude are shown in Fig. 4A. The mean of the relative magnitude of the responses to the 63 peptides under cART was 26.2% of that at pre-cART. The mean of the relative magnitude of the responses to HLA-restricted epitopes was in the order of HLA-B-restricted > HLA-A-restricted > HLA-C-restricted epitope peptides (Fig. 4B), while that to epitopes in each HIV-1 protein was in the order of Pol-specific > Gag-specific > Nef-specific > Env/Rev-specific epitope peptides (Fig. 4C).

**TABLE 1:** Relative magnitude of T-cell responses to HIV-1 epitope peptides

HLA	Epitope	No. of tested individuals	% Mean relative T-cell response under cART (range)^*a*^	HLA	Epitope	No. of tested individuals	% Mean relative T-cell response under cART (range)^*a*^
A*11:01	NefQK10	7	14.3 (0–100)	B*40:02	GagTL8^*c*^	12	29.4 (0–100)
	PolQK9	3	0 (0–0)		PolGI8^*c*^	11	44.5 (0–100)
A*02:01 A*02:06 A*02:07	GagAA9^*b*^ ^,*c*^	4	25.0 (0–100)	B*40:06	PolGA9^*c*^	11	49.5 (0–100)
	GagYL9	6	44.2 (0–100)		PolIT10	11	21.0 (0–92.3)
	PolIV10	8	28.8 (0–100)		PolLA9	8	23.6 (0–100)
	PolSV9	13	26.7 (0–100)	B*48:01	GagGI8	3	33.3 (0–100)
	PolVV11	13	21.4 (0–100)		GagRI9	2	50.0 (0–100)
A*24:02	EnvRI11	18	24.5 (0–100)		GagVL9	1	0 (0–0)
	EnvRL9	22	20.2 (0–100)	B*51:01	EnvLI9	2	50.0 (0–100)
	EnvWI9	5	40.0 (0–100)		GagNI9	4	50.0 (0–100)
	GagKW9	6	66.7 (0–100)		PolLI9^*c*^	13	30.3 (0–100)
	NefRF10	26	24.7 (0–100)		PolTI8^*c*^	4	49.8 (20-100)
A*26:01 A*26:02 A*26:03	EnvEA10	3	14.0 (0–41.9)		RevVL11	1	100 (100–100)
	GagEL9	11	44.5 (0–100)	B*52:01	GagMI8^*c*^	7	0 (0–0)
	GagTM9	5	17.9 (0–70.6)		GagRI8^*c*^	12	22.8 (0–100)
	PolEY9	4	30.5 (0–100)		GagWV8^*c*^	10	16.7 (0–100)
A*31:01	GagIK10	8	2.3 (0–18.3)		PolLI8	8	36.1 (0–100)
A*33:03	GagHR10^*c*^	2	16.2 (0–32.4)		PolSI8^*c*^	10	24.2 (0–100)
	PolER10^*c*^	5	24.2 (0–52.4)	B*54:01	PolFP10	4	25.0 (0–100)
	PolFR9	2	0 (0–0)		PolFV11	4	25.0 (0–100)
B*15:01	GagGY9	10	28.1 (0–0)		PolFV9	4	25.0 (0–100)
	PolIQY11	5	0 (0–100)	B*67:01	GagTL9^*c*^	1	0 (0–0)
B*35:01	EnvDL9	4	8.0 (0–18.4)	C*08:01 C*08:03	GagKL9	7	31.7 (0–76.6)
	NefFL9	6	53.1 (0–100)		PolIL8	8	43.6 (0–100)
	NefRY11	9	19.6 (0–100)	C*12:02	EnvRL9	14	8.7 (0–100)
	NefVY8	7	25.6 (0–100)		NefMY9^*c*^	4	48.1 (0–100)
	NefYF9	12	29.4 (0–100)		PolIY11	7	14.3 (0–100)
	PolEY10	8	60.6 (12.5–100)	C*14:02 C*14:03	GagAM9	10	17.1 (0–100)
	PolTY9	6	39.0 (0–100)		GagLY9	6	0 (0–0)
	PolVY10	9	0.9 (0–7.9)		NefIF8	7	8.4 (0–29.7)
B*39:01	PolEA10	2	50.0 (0–100)		NefYT9	11	19.6 (0–100)
	PolTL9	2	9.5 (0–19)				

^*a*^ Spot-forming units (SFUs) for each HIV-1 epitope peptide under cART / pre-cART × 100.

^*b*^ All tested individuals carry HLA-A*02:06 allele.

^*c*^ Protective epitope. GagAA9 was reported as HLA-A*02:06-restricted protective epitope.

### Effect of long-term cART on the breadth of T-cell responses to HIV-1 epitope peptides at the donor level

**Fig 5:**
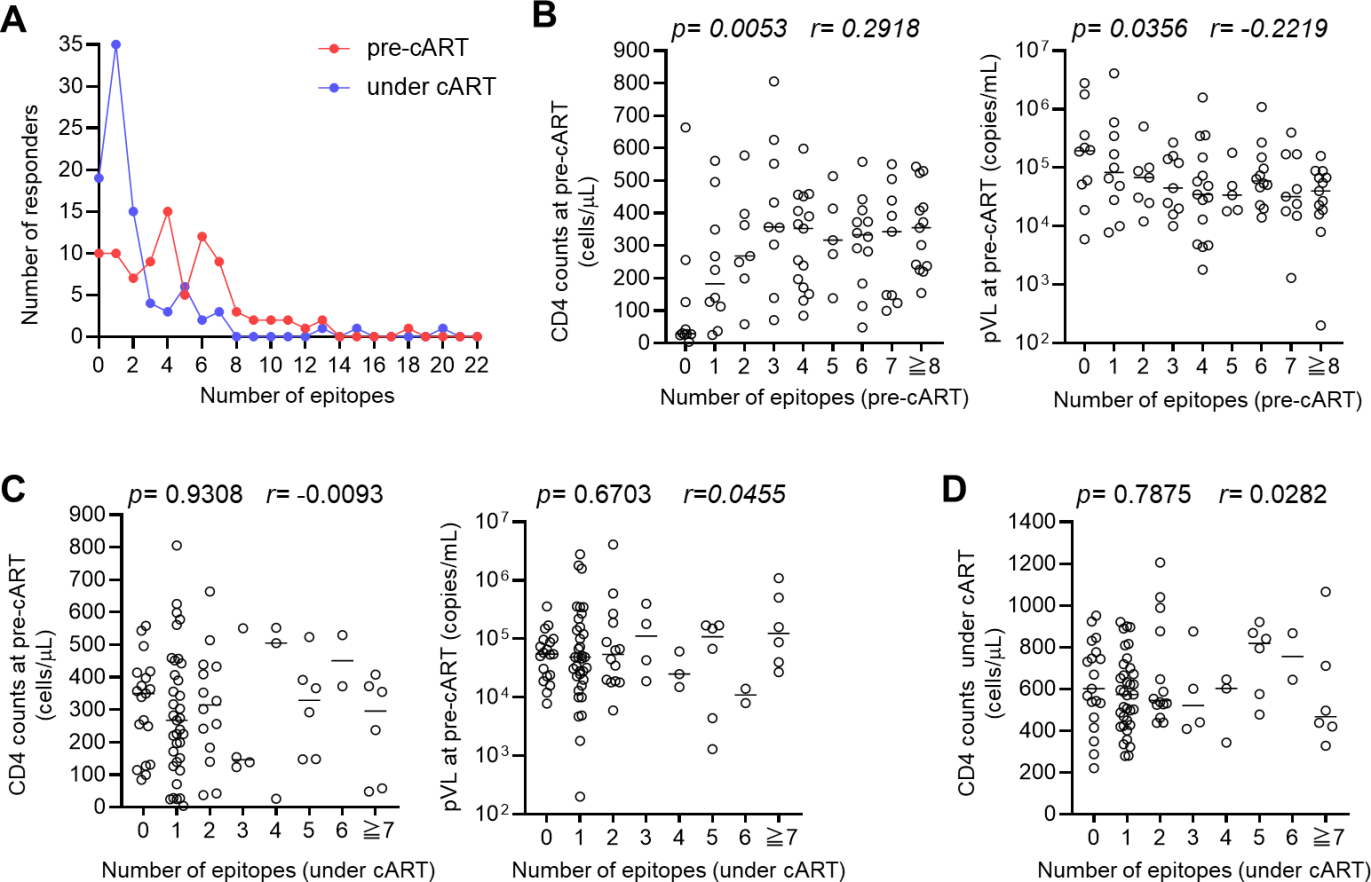
Effect of long-term cART on the breadth of T-cell responses under cART and correlation between the breadth of T-cell responses and clinical outcome. (**A**) Number of responders to HIV-1 epitope peptides in 90 HIV-1-infected individuals at pre-cART and under cART. The median breadths at pre-cART and under cART are 4 and 1, respectively. Statistical analyses performed by Mann-Whitney test showed a significant difference between them (*P* = 2 × 10^−8^). (**B**) Correlation between the breadth of T-cell responses to epitope peptides at pre-cART and CD4 count or pVL at pre-cART. (**C**) Correlation between the breadth of T-cell responses to epitope peptides under cART and CD4 count or pVL at pre-cART. (**D**). Correlation between the breadth of T-cell responses to epitope peptides under cART and CD4 count under cART. Horizontal bars in each figure represent the median CD4 count or pVL. Statistical analysis was performed using Spearman’s rank correlation test (B–D).

We analyzed the effect of long-term cART on the breadth of T-cell responses to HIV-1 epitope peptides at the donor level. The breadth of T-cell responses to epitope peptides at pre-cART was compared with that under cART (Fig. 5A). The breadth under cART was significantly reduced compared with that at pre-cART (the median breadths at pre-cART and under cART are 4 and 1, respectively; *P* = 2 × 10^−8^). Next, we analyzed the correlations of the breadth of T-cell responses with clinical outcome, pVL and CD4 T-cell count. The breadth of T-cell responses at pre-cART significantly correlated negatively with pVL and positively with CD4 count at pre-cART (Fig. 5B), indicating that these T cells have the ability to suppress HIV-1 replication at pre-cART. In contrast, the breadth of the T-cell responses under cART did not correlate with clinical outcome, pVL or CD4 T-cell count, at pre-cART (Fig. 5C), suggesting that clinical outcome at pre-cART does not affect the T-cell response at the donor level. Finally, we analyzed the correlation between the breadth of T-cell responses under cART and CD4 T-cell count under cART. The breadth of the T-cell responses under cART did not correlate with CD4 T-cell count under cART (Fig. 5D). These results suggest that, at the donor level, the T-cell responses under cART do not influence CD4 T-cell count under cART or vice versa.

### T-cell responses to HIV-1 protective epitope peptides under cART

**Fig 6:**
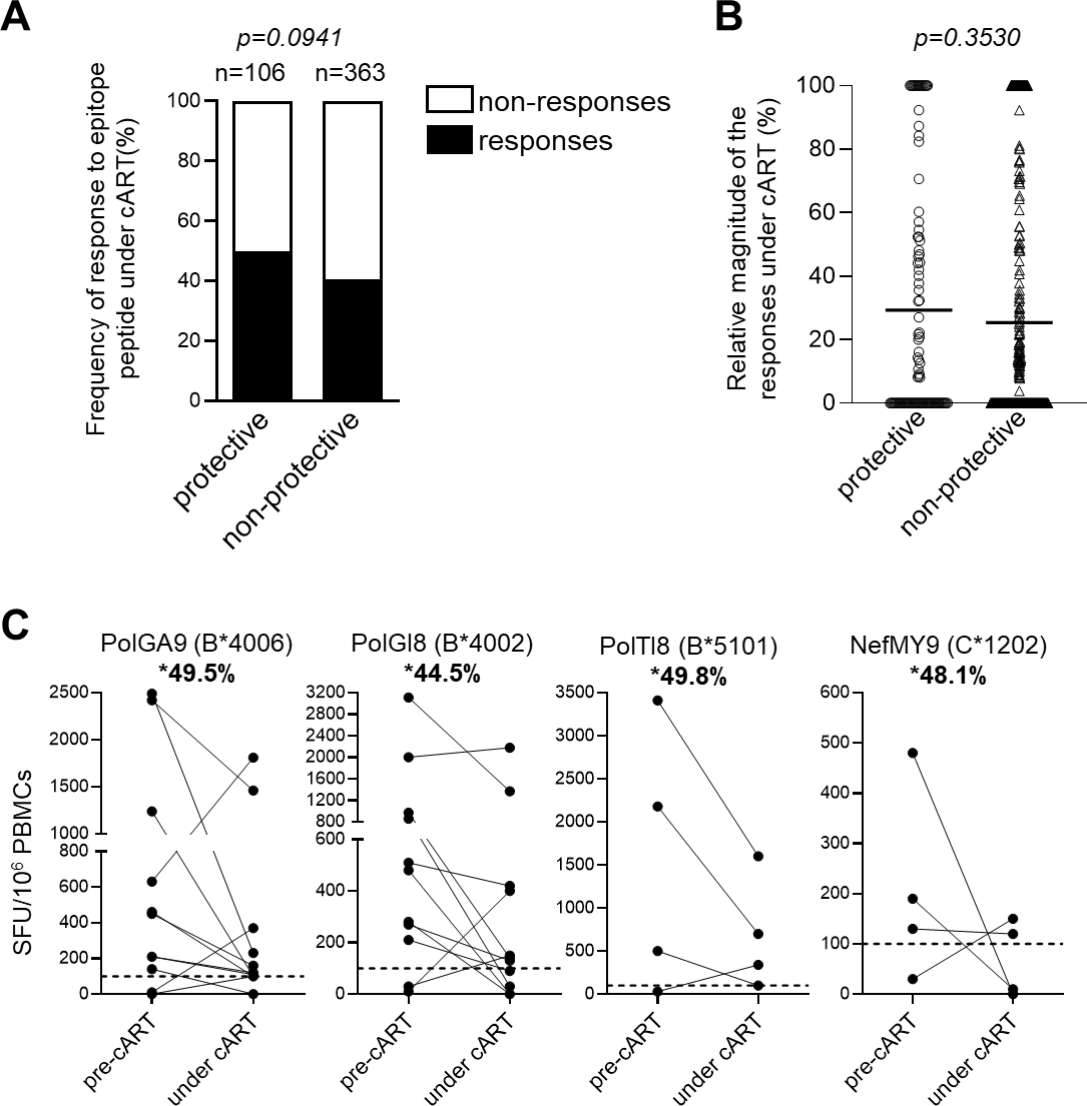
Frequency of responders and magnitude of responses to HIV-1 protective epitope peptides under cART (**A**). The frequency of positive responses to HIV-1 protective epitope peptides or to HIV-1 non-protective epitope peptides under cART. The frequency of positive responses, which was calculated as shown in Fig. 2 legend. The frequency of positive responses between protective and non-protective epitopes was analyzed statistically by Fisher’s exact test. (**B**) Magnitude of T-cell responses to HIV-1 protective epitope peptides or to HIV-1 non-protective epitope peptides under cART versus at pre-cART. Horizontal bars in each figure represent mean magnitude of T-cell responses under cART versus at pre-cART. Statistical analysis was performed using two-tailed unpaired *t*-test. (**C**) Representative cases of positive T-cell responses to protective epitope peptides at pre-cART and under cART. *Percentages of SFU under cART versus that at pre-cART are presented in each figure.

CD8^+^ T cells specific for some HIV-1 epitopes, which are called protective epitopes, have the ability to effectively suppress HIV-1 in treatment-naïve PLWH (39, 40, 42, 45, 47, 48). It is expected that the protective epitope-specific CD8^+^ T cells are effectively maintained even under a situation of weak antigen presentation such as long-term cART because they might have the ability to recognize HIV-1 epitopes more than CD8^+^ T cells specific for non-protective epitopes. We compared the frequency of the protective epitope-specific CD8^+^ T cells under cART with that of non-protective epitope-specific ones. Overall, 14 of 63 epitopes tested were previously reported as protective epitopes (Table S1 in supplemental material). The frequency of positive responses to these protective epitopes under cART showed a trend of being higher than that to non-protective epitopes (50.0% versus 40.4%; *P* = 0.09) (Fig. 6A). The relative magnitude of the response to 14 protective epitope peptides is presented in Table 1. There was a small difference in the relative magnitude of the response between all 14 protective epitopes and 49 non-protective epitopes (29.3% versus 25.3%) (Fig. 6B). These findings suggest that the protective epitope-specific CD8^+^ T cells might be maintained under cART more than the non-protective epitope-specific ones. The relative magnitude of the response to four protective epitope peptides under cART was >40% (Fig. 6C), implying that these T cells might be expected to be effector T cells for curative treatment.

### Minimum effect of clinical outcome at pre-cART on HIV-1-specific T-cell responses under cART

**Fig 7:**
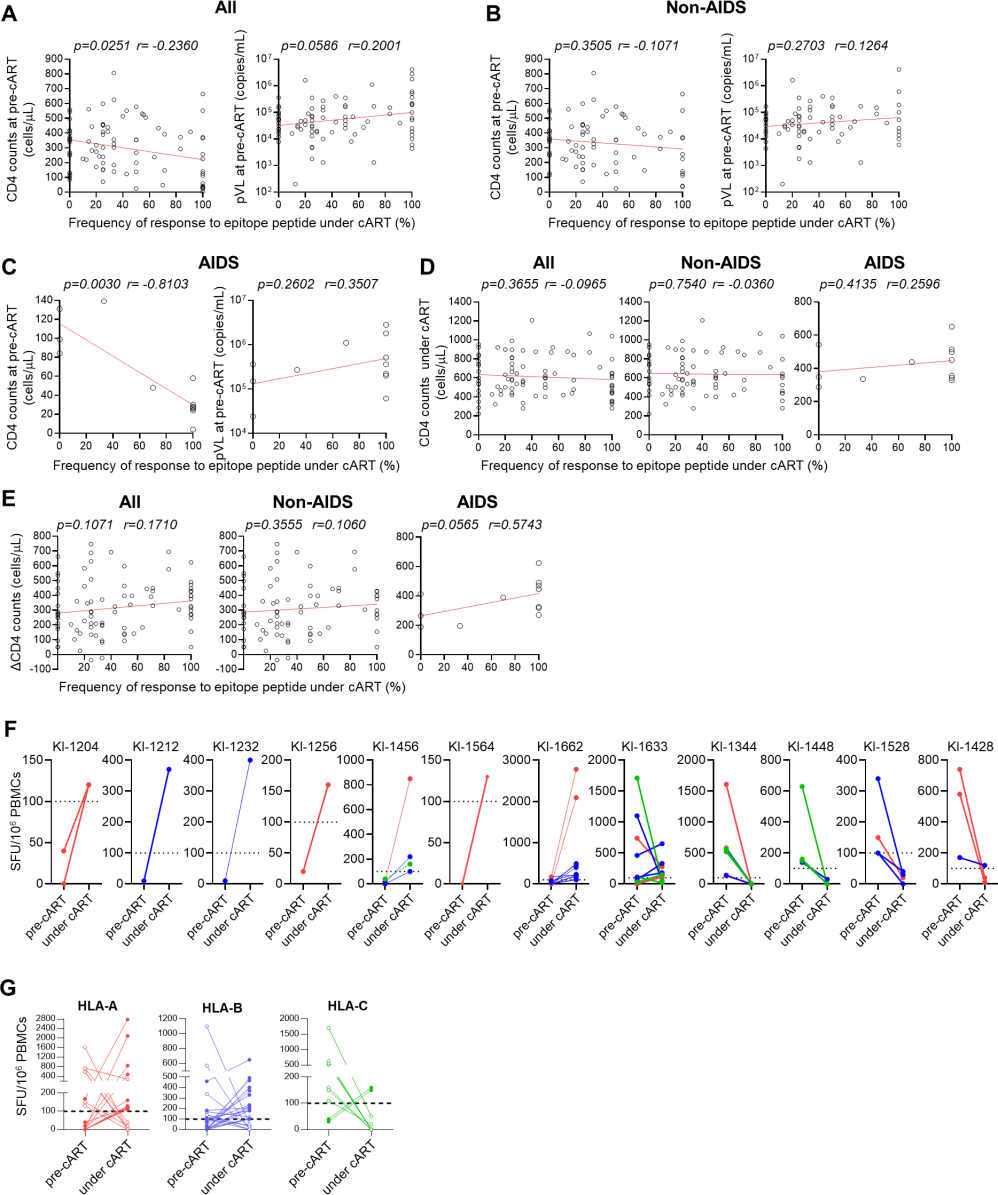
Recovery of HIV-1-specific T-cell induction in AIDS patients under cART (**A–C**). Correlation between the frequency of the T-cell responses under cART and CD4 count or pVL at pre-cART in all 90 individuals (**A**), in 78 non-AIDS HIV patients (**B**), and in 12 AIDS patients (**C**). (**D**) Correlation between the frequency of the T-cell responses under cART and CD4 count under cART. (**E**) Correlation between the frequency of the T-cell responses under cART and ΔCD4 (CD4 count under cART minus CD4 count at pre-cART) in all 90 individuals, 78 non-AIDS HIV patients, and 12 AIDS patients. The correlations were statistically analyzed by Spearman’s correlation test. Each dot represents the frequency of positive T-cell responses to all epitope peptides tested under cART in each individual. The frequency of T-cell responses to epitope peptides under cART was calculated as shown in Fig. 2 legend. (**F**) T-cell responses at pre-cART and under cART in 12 AIDS patients. Red, blue, and green lines represent T-cell responses to HLA-A-restricted, HLA-B-restricted, and HLA-C-restricted epitope peptides, respectively. (**G**) T-cell responses to HLA-A-restricted, HLA-B-restricted, and HLA-C-restricted epitope peptides at pre-cART and under cART in 12 AIDS patients. The dotted line at 100 SFU/10^6^ PBMCs indicates the threshold for a positive response.

We investigated the effect of clinical outcome at pre-cART on HIV-1-specific T-cell responses under cART. We analyzed the correlation between CD4 count or pVL at pre-cART and the frequency of positive T-cell responses to epitopes under cART in 90 individuals, including 78 non-AIDS HIV-1 patients and 12 AIDS patients. There was a significant negative correlation between CD4 count at pre-cART and the frequency of positive T-cell responses under cART, along with a positive but not significant correlation between pVL at pre-cART and the frequency of positive T-cell responses under cART (Fig. 7A). These correlations were not found in 78 non-AIDS HIV patients (Fig. 7B). These results together suggest that the effect of clinical outcome at pre-cART on T-cell responses to epitopes under cART is minimal; however, they imply that there is a negative correlation between CD4 count at pre-cART and the frequency of positive T-cell responses under cART in individuals with a worse clinical outcome, such as AIDS patients.

### Recovery of HIV-1-specific T-cell responses under cART in AIDS patients

Next, we analyzed the correlation between CD4 count or pVL at pre-cART and the frequency of T-cell responses to HIV-1 epitopes under cART in 12 AIDS patients (Table S3 in supplemental material). The results showed a significant negative correlation between CD4 count at pre-cART and the frequency of positive T-cell responses under cART in 12 AIDS patients (Fig. 7C), suggesting that HIV-1-specific T cells were effectively elicited under cART in AIDS patients with a lower CD4 count at pre-ART. No significant difference was found between CD4 count under cART and the frequency of positive T-cell responses under cART in all 90 individuals, non-AIDS HIV-1 patients, and AIDS patients (Fig. 7D), whereas a trend of a positive association of ΔCD4 (CD4 count under cART minus CD4 count at pre-cART) with the frequency of positive T-cell responses under cART was found in AIDS patients (Fig. 7E). These results suggest that AIDS patients who recovered more immunologically as a result of cART obtained the ability to elicit HIV-1-specific T-cell responses.

HIV-1-specific T cells were not elicited under cART in three patients (KI-1344, KI-1448, and KI-1528), while one of three HIV-1-specific T cells was elicited in KI-1428 (Fig. 7F). Three of these four patients had a lower ΔCD4 count than the other AIDS patients who had a higher number of HIV-1-specific T cells under cART (Fig. 7E). These results suggest that HIV-1-specific T cells are not effectively induced if there is only partial immunological recovery as a result of cART. In contrast, eight AIDS patients revealed higher T-cell responses to HIV-1 epitopes under cART than those at pre-cART (Fig. 7F).

We analyzed the magnitude of HLA-A-restricted, HLA-B-restricted, or HLA-C-restricted T-cell responses under cART in AIDS patients. Higher number of spot-forming units (SFU) was found under cART than at pre-cART in 11 of 18 HLA-A-restricted T-cell responses (61.1%) and 16 of 24 HLA-B-restricted T-cell ones (66.7%), whereas it was found in only two of nine HLA-C-restricted T-cell responses (22.2%) (Fig. 7G). These results imply that HLA-C-restricted T cells had less ability to recover after long-term cART than the HLA-A-restricted and HLA-B-restricted ones.

**Fig 8:**
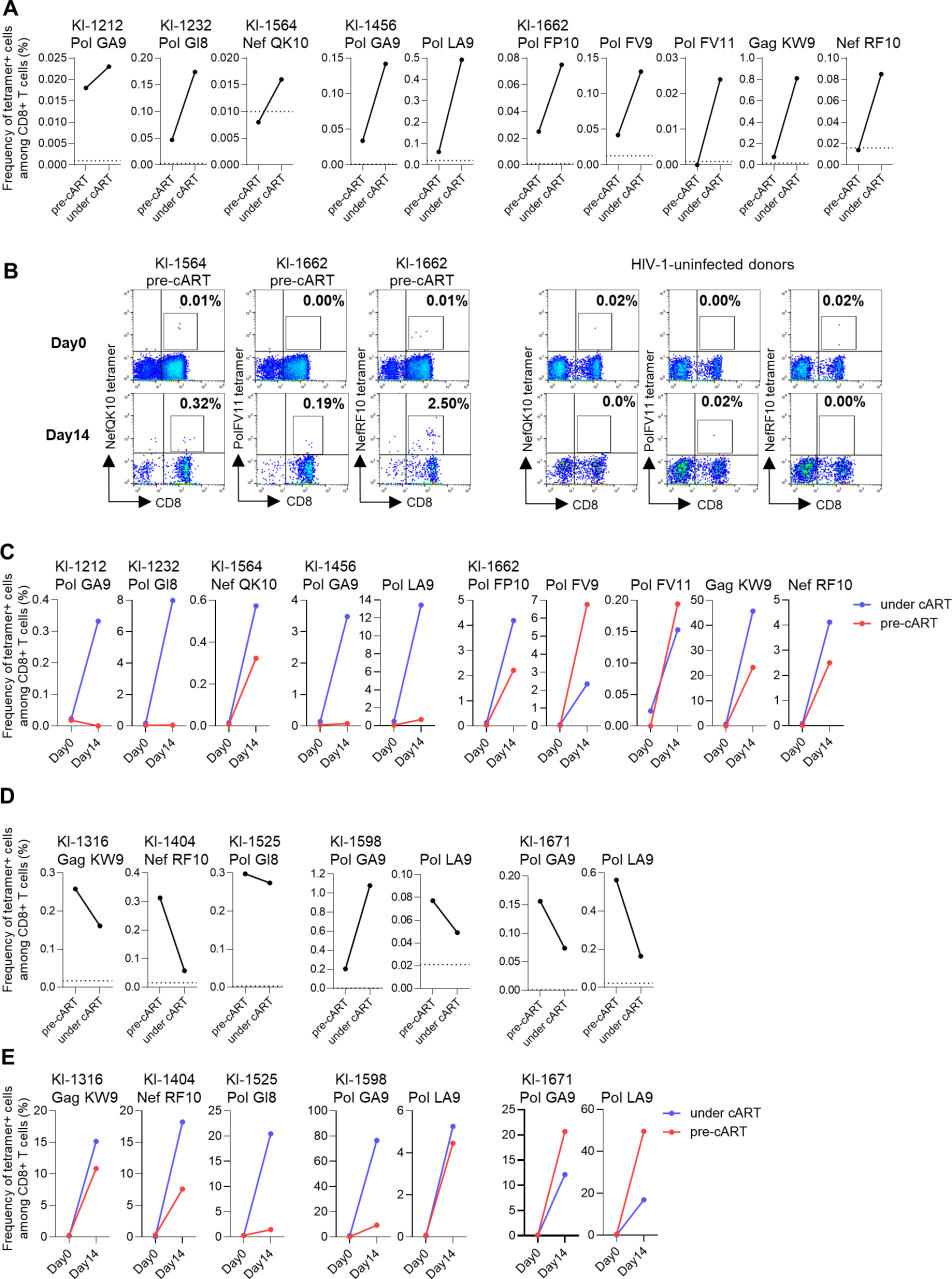
Identification of HIV-1-specific CD8^+^ T cells by using HLA tetramers and their ability to proliferate (**A**). Detection of HIV-1-specific CD8^+^ T cells at pre-cART and under cART in *ex vivo* PBMCs from five AIDS patients by using nine HLA-peptide tetramers. The dotted line represents the threshold for positive binding. (**B**) Proliferative ability of three HIV-1-specific T cells that were not found among PBMCs collected at pre-cART. HIV-1-specific T cells at pre-cART among *ex vivo* PBMCs (day 0) and the cultured T cells stimulated with epitope peptide (day 14) were detected by using the specific HLA-peptide tetramers (HLA-A*11:01-NefQK10, HLA-A*24:02-NefRF10, and HLA-B*54:01-PolFV11). The frequency of the tetramer^+^ cells among the CD8^+^ T cells is indicated. (**C**) Proliferative ability of nine HIV-1-specific T cells at pre-cART and under cART in AIDS patients. (**D**) Identification of HIV-1-specific CD8^+^ T cells specific for five epitopes in five non-AIDS HIV patients. Five HLA-peptide tetramers were used to detect the specific CD8^+^ T cells in PBMCs from five patients. (**E**) Proliferative ability of five HIV-1-specific T cells at pre-cART and under cART in non-AIDS HIV patients.

To confirm the induction of HIV-specific T cells in AIDS patients, we sought to detect these HIV-specific T cells by using the HLA tetramers. We generated nine tetramers (HLA-B*40:06-PolGA9 or -PolLA9 and HLA-B*40:02-PolGI8, HLA-A*24:02-NefRF10 or -GagKW9, HLA-B*54:01-PolFP10, -PolFV9, or -PolFV11, HLA-A*11:01-NefQK10) and selected five individuals in whom HIV-1-specific T cells were detected under cART but not at pre-cART by using *ex vivo* ELISpot assay. CD8^+^ T cells specific for nine HIV-1 epitopes were detected in these individuals (Fig. 8A). Interestingly, CD8^+^ T cells specific for three epitopes (NefQK10, NefRF10, and PolFV11) were not detected at pre-cART in two individuals (KI-1564 and KI-1662), whereas CD8^+^ T cells specific for other epitopes were found in four individuals. Thus, HIV-specific T cells were detected at pre-cART in most AIDS patients in the analysis using the tetramers, whereas they were still not detected in some cases (NefQK10-specific T cells in KI-1564 and NefRF10-specific and PolFV11-specific T cells in KI-1662). These results confirmed that the frequency of HIV-1-specific T cells was much higher under cART than that at pre-cART in AIDS patients.

### Proliferative ability of HIV-1-specific T cells *in vitro*

We next investigated the ability of HIV-1-specific T cells to proliferate after stimulation with epitope peptides. PBMCs at pre-cART and under cART were stimulated with the corresponding epitope peptides and then cultured for 14 days. We analyzed CD8^+^ T cells specific for nine epitopes in five AIDS patients by using the tetramers. In the culture of PBMCs collected at pre-cART (day 14), we found CD8^+^ T cells specific for NefQK10 in KI-1564 and those specific for NefRF10 or PolFV11 in KI-1662, although these T cells were not detected among *ex vivo* PBMCs at pre-cART (day 0) in these individuals (Fig. 8B). These findings confirmed that these T cells existed in PBMCs collected from these AIDS patients at pre-cART. In contrast, T cells specific for PolGA9 in KI-1212 and KI-1456 and those specific for PolGI8 in KI-1232 were not detected after *in vitro* culture of PBMCs collected at pre-cART, although these were detected in PBMCs collected at pre-cART (Fig. 8C), indicating that these T cells lost the ability to proliferate at pre-ART. In the culture of PBMCs collected under cART, CD8^+^ T cells specific for all nine epitopes were detected in five individuals (Fig. 8C), indicating that these T cells recovered their proliferative ability after cART for more than 2 years.

We also investigated the ability of HIV-1-specific T cells to proliferate in non-AIDS HIV patients and compared it to that in AIDS patients. We selected CD8^+^ T cells specific for five epitopes that were analyzed in the AIDS patients. Except for CD8^+^ T cells specific for PolGA9 in KI-1598, the frequency of T cells specific for other epitopes in PBMCs collected under cART was lower than that in PBMCs collected at pre-cART (Fig. 8D). Meanwhile, the frequency of CD8^+^ T cells specific for all epitopes in culture of PBMCs collected at pre-cART and under cART was higher than that before the culture (Fig. 8E), indicating that HIV-1-specific T cells at pre-cART and under cART had the ability to proliferate *in vitro* in these non-AIDS HIV patients. These results together indicate that HIV-1-specific T cells had the ability to proliferate in both AIDS patients and non-AIDS HIV patients after cART for more than 2 years.

## DISCUSSION

In the present study, we investigated T cells specific for 63 HIV-1 epitopes in chronically HIV-1-infected individuals who had received cART for >2 years and compared the frequency of HIV-1-specific T cells under cART to that at pre-cART within the same individuals. We demonstrated T-cell responses to 56 HIV-1 epitopes under cART in 90 HIV-1-infected individuals. Approximately 42% of HIV-1-specific T-cell responses were found under cART, while the magnitude of HIV-1-specific T-cell responses under cART was reduced to 26% of the T-cell responses at pre-cART. Thus, half of HIV-1-specific CD8^+^ T cells were maintained during long-term cART, which reflects conditions of weak antigen presentation. The effect of long-term cART on HIV-1-specific CD8^+^ T cells was epitope-dependent. Interestingly, the rate and the intensity of positive T-cell responses to HLA-B-restricted epitopes under cART were higher than those to HLA-A-restricted or HLA-C-restricted ones. These results suggest higher responsiveness of T cells specific to HLA-B-restricted epitopes than that of T cells specific to HLA-A-restricted or HLA-C-restricted epitopes. Another possible explanation is that HLA-B-restricted epitopes may be presented more effectively than HLA-A-restricted or HLA-C-restricted ones. Indeed, the number of identified HIV-1 epitopes in HLA-B is known to be much higher than that in HLA-A or HLA-C (https://www.hiv.lanl.gov/content/index).

Previous studies showed that treatment-naïve individuals having T cells specific for a given epitope had significantly better clinical outcomes than those without them (39, 40, 42, 45, 47, 48). T cells specific for these epitopes, called protective epitopes, have a higher capacity to suppress the replication of HIV-1 than other HIV-1-specific T cells. T cells specific for protective epitopes are thus expected to have a stronger ability to reduce reservoir size than other HIV-1-specific T cells. In the present study, we demonstrated that CD8^+^ T cells specific for 12 protective epitopes are still maintained or elicited after long-term cART and showed a trend of higher frequency of responses to these protective epitopes under cART than that of responses to non-protective epitopes. These findings imply the possibility that protective epitope-specific T cells are useful as effector T cells to eradicate latent reservoirs in curative treatment using the “shock and kill” approach. However, further analysis of T cells specific for protective epitopes under cART in a larger-scale cohort is necessary for the development of a curative treatment using these T cells.

HIV-1-specific T cells were frequently detected after long-term cART in AIDS patients with a lower CD4 count at pre-cART, while there was a tendency for a positive association of ΔCD4 with the frequency of positive T-cell responses under cART. These findings suggest that AIDS patients had the ability to elicit or expand HIV-1-specific T cells if the recovery of immunological condition was achieved via long-term cART. Analyses using both *ex vivo* ELISpot assay and flow cytometry with HLA tetramers demonstrated that HIV-1-specific T cells were not detected or only a very small number of them were detected at pre-cART in AIDS patients, although these T cells were detected under cART. Even though HIV-1-specific T cells specific for three epitopes (HLA-A*11:01-NefQK10, HLA-A*24:02-NefRF10, and HLA-B*54:01-PolFV11) were not detected by both assays in PBMCs collected at pre-cART from two AIDS patients, these T cells were induced after these PBMCs were stimulated with epitope peptides *in vitro*. These findings together suggested that T cells specific for all nine epitopes were very weakly elicited at pre-cART in AIDS patients. These T cells were detected in *ex vivo* PBMCs collected under cART from these patients and effectively proliferated after *in vitro* stimulation with the epitope peptides, indicating that long-term cART restored the ability of HIV-1-specific T cells to proliferate *in vivo* in AIDS patients.

Previous studies showed that HIV-1-specific T cells at the acute infection phase have a stronger capacity to recognize HIV-1-infected cells than those in the chronic infection phase (49, 50) and that ART initiation in acute HIV infection preserves functional HIV-specific CD8^+^ T cells (7, 51, 52). However, because PLWH who initiated cART at the acute infection phase are a minority among those receiving long-term cART, methods that restore the ability of HIV-1-specific CD8^+^ T cells should be developed to eradicate latent reservoirs in PLWH who initiated cART at the chronic infection phase. The present study demonstrated that approximately 42% of HIV-1-specific T cells and 50% of HIV-1 protective epitope-specific T cells detected at pre-cART were found under cART and that the induction of HIV-1-specific T cells was restored by long-term cART in AIDS patients, along with the ability of these T cells to proliferate *in vitro*. Therefore, these highly detectable HIV-1-specific T cells, especially protective epitope-specific T cells, might be candidates as effector T cells in ‘‘shock and kill’’ therapy in both AIDS patients and non-AIDS HIV patients. Further studies of these T cells are expected to clarify which epitope-specific T cells can contribute to eradicating latent reservoirs during cART. Such studies could then provide ideas for novel methods to enhance the ability of these CD8^+^ T cells during cART.

In the present study, we clarified the effect of long-term cART on large numbers of HIV-1-specific CD8^+^ T cells. The frequency of responders to these HIV-1 epitopes under cART varied from 0% to 100%. These findings suggest that the maintenance of HIV-1-specific CD8^+^ T cells is epitope-dependent under conditions of weak antigen presentation for a long time. Further analysis of CD8^+^ T cells specific for large numbers of HIV-1 epitopes will contribute to understanding not only the immune control of HIV-1-infected cells under cART but also the maintenance of HIV-1-specific CD8^+^ T cells under conditions of weak antigen presentation.

## MATERIALS AND METHODS

### Subjects

Ninety-six individuals infected with HIV-1 subtype B, who were treated with cART from chronic infection for more than 2 years, were recruited in the National Center for Global Health and Medicine, Tokyo, Japan. HIV-1-infected individuals who had opportunistic diseases caused by HIV-induced immunodeficiency at the initiation of cART are defined as AIDS patients, while HIV-1-infected asymptomatic carriers at initiation of cART were defined as non-AIDS patients. PBMCs were separated from whole blood of these individuals within 6 months (0–6 months, median 0 months) before cART initiation and at more than 2 years (2–8 years, median 6 years) after cART initiation. All individuals had maintained undetectable pVL (plasma HIV RNA levels <20 copies per milliliter) for 2 years at least since initiation of cART and at all subsequent time points.

### HLA genotyping

HLA-A, HLA-B, and HLA-C genotypes were identified by the Luminex microbead method (Luminex 100 system; Luminex Corporate, Austin, TX, USA) at the NPO HLA Laboratory (Kyoto, Japan). They were reported according to the nomenclature of the HLA Dictionary (53)

### HIV-1 T-cell epitope peptides

T-cell epitope peptides were synthesized using an automated multiple-peptide synthesizer and purified by high-performance liquid chromatography. Peptides with more than 90% purity were used in this study.

### IFN-γ ELISpot assay

ELISpot assays were performed as previously described (40). Briefly, 1  ×  10^5^ PBMCs from each individual and a 1 µM concentration of each HIV-1 peptide were plated into 96-well polyvinylidene plates (Millipore) that had been coated overnight with 5 µg/mL anti-IFN-γ (interferon-gamma) mAb(monoclonal antibody) 1-D1K (Mabtech). The plates were then incubated for 16 h at 37°C and then the IFN-γ-producing cells were detected as previously described in detail (40). The spots were counted with an Eliphoto-Counter instrument (Minerva Tech). The number of spots was calculated per 10^6^ PBMCs; 100 spots/10^6^ PBMCs was defined as a positive response. The mean values +5 SD of the SFUs of samples from 12 HIV-1-naïve individuals for the peptide pool were 46 SFU/10^6^ PBMCs in a previous study (42). Therefore, we defined a positive ELISpot response as larger than 100 SFU/10^6^ PBMCs to exclude false positive.

### Detection of HIV-1-specific T cells using the tetramers

HLA-B*40:06-PolGA9 or -PolLA9 peptide tetrameric complexes (tetramers) and HLA-B*40:02-PolGI8, HLA-A*24:02-NefRF10 or -GagKW9, HLA-B*54:01-PolFP10, -PolFV9, or -PolFV11, HLA-A*11:01-NefQK10 complexes were generated as previously described (33, 37, 38, 45). PBMCs or the bulk T cells were stained with phycoerythrin-conjugated epitope-specific tetramers at 37°C for 30 min. The cells were then washed twice with RPMI-1640 medium containing 5% fetal bovine serum (FBS) (R5), followed by staining with allophycocyanin-conjugated anti-CD8 mAb (BioLegend) and the reagents of a LIVE/DEAD Fixable Near-IR Dead Cell Stain kit (Invitrogen) at 4°C for 30 min. Finally, the cells were washed twice with R5 and then analyzed using a FACS CantoII. We calculated means  + 2 SD of the frequency of tetramer-binding CD8^+^ T cells from three HIV-1-negative individuals for each epitope and then defined a positive frequency of NefRF10, GagKW9, NefQK10, PolLA9, PolGA9, PolGI8, PolFV11, PolFV9, and PolFP10-tetramer-binding CD8^+^ T cells as 0.016%, 0.017%, 0.010%, 0.021%, 0.000%, 0.003%, 0.000%, 0.000%, and 0.013%, respectively.

### Proliferative capacity of epitope-specific CD8^+^ T cells

After cryopreserved PBMCs were thawed, 1 ×  10^6^ PBMCs were stained with HIV-1 epitope-specific tetramers to evaluate the frequency of the epitope-specific CD8^+^ T cells on day 0. PBMCs were plated at a concentration of 1 ×  10^6^ cells per well in 200 µL of RPMI-1640 medium (Thermo Fisher Scientific) supplemented with 10% FBS, 1× MEM nonessential amino acid solution (Gibco), 1 mM sodium pyruvate solution, and 20 ng/mL human recombinant interleukin 2 (ProSpec), and then stimulated with 100 nM epitope peptides. After 14 days in culture, the bulk T cells were stained with the epitope-specific tetramers. The proliferative capacity of HIV-1 epitope-specific CD8^+^ T cells was evaluated based on the frequency of tetramer^+^ cells among total CD8^+^ T cells on day 14 versus that on day 0.

### Statistics

Statistical analyses were performed using GraphPad Prism 8. Groups were compared by performing two-tailed unpaired *t*-test or Mann-Whitney U-tests. The frequency of T-cell responses was statistically compared between protective and non-protective epitopes using Fisher’s exact test. Correlations were determined by Spearman’s rank test. *P*-values of <0.05 were considered significant.

## SUPPLEMENTAL MATERIAL

Table S1 and Table S3 jvi.01024-23-s0001.docx Table S1 (HIV-1 CD8^＋^T cell epitopes used in the present study) and Table S3 (Information for clinical data in AIDS patients).

Table S2 jvi.01024-23-s0002.xlsx T-cell responses to HIV-1 epitope peptides at pre-cART and under cART.

ASM does not own the copyrights to Supplemental Material that may be linked to, or accessed through, an article. The authors have granted ASM a non-exclusive, world-wide license to publish the Supplemental Material files. Please contact the corresponding author directly for reuse.
