# Supplementary material for: Epitope-dependent effect of long-term cART on maintenance and recovery of HIV-1-specific CD8+ T cells
Source: J Virol. 2023 Oct 25;97(11):e01024-23. doi: 10.1128/jvi.01024-23 (PMC10688310; doi:10.1128/jvi.01024-23)
Supplement: Table S1 and Table S3 — Table S1 (HIV-1 CD8＋T cell epitopes used in the present study) and Table S3 (Information for clinical data in AIDS patients). [file jvi.01024-23-s0001.docx]

**Supplemental Table 1. HIV-1 CD8^＋^T cell epitopes used in the present study**

| **HLA** | **Epitope** | **Sequences** | **Reference** |
| --- | --- | --- | --- |
| A*11:01 | Nef AK9 | AVDLSHFLK | 34 |
| A*11:01 | Nef QK10 | QVPLRPMTYK | 34 |
| A*11:01 | Pol QK9 | QIYAGIKVK | 27 |
| A*11:01 | Env SK9 | SVITQACPK | 27 |
| A*02:01/A*02:06/A*02:07 | Gag AA9^a, c^ | ATLEEMMTA | 39, 42 |
| A*02:01/A*02:06/A*02:07 | Gag YL9^a^ | YVDRFYKTL | 42 |
| A*02:01/A*02:06/A*02:07 | Pol IV10^a^ | IYQYMDDLYV | 45 |
| A*02:01/A*02:06/A*02:07 | Pol SV9^a^ | SQIYAGIKV | 39 |
| A*02:01/A*02:06/A*02:07 | Pol VV11^a^ | VIYQYMDDLYV | 45 |
| A*24:02 | Env RI11 | RYLRDQQLLGI | 21 |
| A*24:02 | Env RL9 | RYLRDQQLL | 21 |
| A*24:02 | Env FF9 | FYCNTTQLF | 21 |
| A*24:02 | Env WI9 | WYIKIFIMI | 26 |
| A*24:02 | Gag KW9 | KYKLKHIVW | 23, 33 |
| A*24:02 | Env RL8 | RYLRDQQL | 21 |
| A*24:02 | Nef RW8 | RYPLTFGW | 38 |
| A*24:02 | Nef RF10 | RYPLTFGWCF | 21, 38 |
| A*26:01/A*26:02/A*26:03 | Env EA10^b^ | EVHNVWATHA | 45 |
| A*26:01/A*26:02/A*26:03 | Gag EL9 | EVIPMFSAL | 29, 31 |
| A*26:01/A*26:02/A*26:03 | Gag TM9^b^ | TLQEQIGWM | 42 |
| A*26:01/A*26:02/A*26:03 | Pol EY9^b^ | ETKLGKAGY | 29 |
| A*31:01 | Nef KR9 | KLAFHHMAR | 30 |
| A*31:01 | Gag IK10 | IAKNCRAPRK | 42 |
| A*33:03 | Gag HR10^c^ | HIAKNCRAPR | 42 |
| A*33:03 | Gag MR9 | MVHQAISPR | 28 |
| A*33:03 | Env VR10 | VFAVLSIVNR | 25 |
| A*33:03 | Env VIR10 | VIEVAQRAYR | 25 |
| A*33:03 | Pol FR9 | FYVDGAANR | 28 |
| A*33:03 | Env ER8 | EVAQRAYR | 25 |
| A*33:03 | Pol TR11 | TLWQRPLVTIR | 28 |
| A*33:03 | Pol ER10^c^ | ELKKIIGQVR | 45 |
| B*15:01 | Gag GY9 | GLNKIVRMY | 42 |
| B*15:01 | Pol IQY11 | IQKQGQGQWTY | 45 |
| B*35:01 | Env DL9 | DPNPQEVVL | 43 |
| **Supplemental Table 1 (Continued…)** | | | |
| **HLA** | **Epitope** | **Sequences** | **Reference** |
| B*35:01 | Nef FL9 | FPVRPQVPL | 22 |
| B*35:01 | Nef RY11 | RPQVPLRPMTY | 43 |
| B*35:01 | Nef VY8 | VPLRPMTY | 43 |
| B*35:01 | Nef YF9 | YPLTFGWCF | 43 |
| B*35:01 | Pol EY10 | EPIVGAETFY | 43 |
| B*35:01 | Pol TY9 | TVLDVGDAY | 43, 45 |
| B*35:01 | Pol NQY9 | NPDIVIYQY | 43, 45 |
| B*35:01 | Pol VY10 | VPLDKDFRKY | 43, 45 |
| B*35:01 | Gag HA9 | HPVHAGPIA | 43 |
| B*39:01 | Pol EA10 | EHLKTAVQMA | 45 |
| B*39:01 | Pol TL9 | THLEGKIIL | 45 |
| B*40:02 | Gag TL8^c^ | TERQANFL | 42 |
| B*40:02 | Pol GI8^c^ | GERIVDII | 39, 45 |
| B*40:06 | Pol GA9^c^ | GERIVDIIA | 45 |
| B*40:06 | Pol IT10 | IEAEVIPAET | 39 |
| B*40:06 | Pol LA9 | LEGKIILVA | 39, 45 |
| B*48:01 | Gag GI8 | VKNWMTETL | 36 |
| B*48:01 | Gag RI9 | GQMVHQAI | 32 |
| B*48:01 | Gag VL9 | RQANFLGKI | 32 |
| B*51:01 | Gag NI9 | NANPDCKTI | 24 |
| B*51:01 | Pol LI9^c^ | LPPVVAKEI | 24 |
| B*51:01 | Pol TI8^c^ | TAFTIPSI | 24, 45 |
| B*51:01 | Rev VL11 | VPLQLPPLERL | 35 |
| B*51:01 | Env RI9 | RAYRAILHI | 24 |
| B*51:01 | Env LI9 | LPCRIKQII | 24 |
| B*51:01 | Pol DL8 | DAYFSVPL | 24 |
| B*51:01 | Gag YI9 | YAPPIGGQI | 24 |
| B*51:01 | Pol QI9 | QGWKGSPAI | 24 |
| B*52:01 | Gag MI8^c^ | MQMLKETI | 39 |
| B*52:01 | Gag RI8^c^ | RMYSPTSI | 39, 42 |
| B*52:01 | Gag WV8^c^ | WMTETLLV | 39, 42 |
| B*52:01 | Pol LI8 | LQKQITKI | 41 |
| B*52:01 | Pol SI8^c^ | SQYALGII | 39 |
| B*54:01 | Pol FP10 | FPISPIETVP | 37 |
| **Supplemental Table 1 (Continued…)** | | | |
| **HLA** | **Epitope** | **Sequences** | **Reference** |
| B*54:01 | Pol FV11 | FPISPIETVPV | 37 |
| B*54:01 | Pol FV9 | FPISPIETV | 37 |
| B*67:01 | Gag NL11 | NPDCKTILKAL | 39, 42 |
| B*67:01 | Gag TL9^c^ | TPQDLNTML | 39 |
| C*08:01/C*08:03 | Gag KL9 | KALGPAATL | 42 |
| C*08:01/C*08:03 | Pol IL8 | IYQYMDDL | 45 |
| C*12:02 | Env RL9 | RAIEAQQHL | 44 |
| C*12:02 | Nef MY9^c^ | MARELHPEY | 40, 44 |
| C*12:02 | Pol IY11 | ILKEPVHGVYY | 41, 44 |
| C*14:02/C*14:03 | Gag AM9 | AFSPEVIPM | 46 |
| C*14:02/C*14:03 | Gag LY9 | LYNTIAVLY | 46 |
| C*14:02/C*14:03 | Nef IF8 | IYHTQGYF | 46 |
| C*14:02/C*14:03 | Nef YT9 | YFPDWQNYT | 46 |

^a^ GagAA9/PolSV9, GagYL9, PolIV10/PolVV11 were reported as HLA-A*02:06-restricted, HLA-A*02:07-restricted, and HLA-A*02:01-restricted epitopes, respectively, but were also analyzed in other 2 HLA-A*02 subtypes^+^ individuals in the present study.

^b^ EnvEA10, GagTM9, PolEY9 were reported as HLA-A*26:03-restricted, HLA-A*26:02-restricted, and HLA-A*26:01-restricted epitope, respectively, but were also analyzed in other 2 HLA-A*26 subtypes^+^ individuals in the present study.

^c^ Protective epitope. GagAA9 was reported as HLA-A*02:06-restricted protective epitope.

**Supplemental Table 3. Information for clinical data in AIDS patients**

| patient ID | pre-cART | | under cART |  |
| --- | --- | --- | --- | --- |
|  | CD4 (cells/μL) | pVL (copies/mL) | CD4 (cells/μL) | ΔCD4^a^ (cells/μL) |
| KI-1204 | 30 | 200000 | 356 | 326 |
| KI-1212 | 4 | 220000 | 450 | 446 |
| KI-1232 | 24 | 1800000 | 514 | 490 |
| KI-1256 | 27 | 2800000 | 498 | 471 |
| KI-1344 | 99 | 24000 | 288 | 189 |
| KI-1428 | 139 | 270000 | 335 | 196 |
| KI-1448 | 131 | 360000 | 543 | 412 |
| KI-1456 | 26 | 61000 | 343 | 317 |
| KI-1528 | 84 | 150000 | 349 | 265 |
| KI-1564 | 28 | 360000 | 651 | 623 |
| KI-1633 | 48 | 1100000 | 438 | 390 |
| KI-1662 | 58 | 510000 | 328 | 270 |

^a^ CD4 count under cART minus CD4 count at pre-cART
